# Notes and News

**Published:** 1895-04-27

**Authors:** 


### Notes and News.

(Collected and Communicated.)

His Royal Highness the Duke of York has consented to become one of the Protectors of the German Hospital, on the occasion of the approaching Jubilee.

St. Thomas's Hospital has received upwards of £22,000 towards the opening of its closed wards. Alderman Sir Stuart Knill has been elected a member of the Grand Committee, and Mr. Alfred John Gibbons has been elected a dispenser at the hospital.

The record for the year 1895 promises to be a bright one amongst the charities. The Press has lately been announcing an unusually large number of legacies and donations. Besides Mr. W. A. Quesdon's bequest of £45,000 to the Hospital Sunday Fund, Mr. H. Sharland leaves £1,000 each to St. Thomas's, Guy's, the Royal Free, St. Luke's, and the Great Ormond Street Hospitals.

In America, the Savannah, Florida, and Western Railway has caused a hospital car to be built for service on the railway. Where an accident occurs far away from skilled medical aid the advantage of such a construction may be imagined, especially as the car is provided with a fully-equipped operating room. In England we are surprisingly badly off for proper travelling arrangements for invalids, even when comparatively convalescent. Ordinary comfort can only be obtained at great expense on some railways, although the trains are largely used by invalids seeking warmer quarters on the South Coast. A warming apparatus, such as is in use on some German lines, which is capable of regulation, would be especially suitable, instead of the meagre and steadily decreasing heat of a foot warmer, which is all that is provided for travellers to Hastings in the draughty carriages of the South-Eastern Railway, and in those which are but little better on the London, Brighton, and South Coast Railway to the same place, although this is one of the most frequented of winter resorts.

The American National Academy of Sciences has awarded a gold medal to Lord Rayleigh for his discovery of argon.

Sir James Reckett has presented £1,000 to the Hull Royal Infirmary. This last munificent donation crowns a series of benefits to the infirmary from the same gentleman.

H.R.H. the Duchess of Teck will, on Tuesday, July 9th, open the new building of the London Homœopathic Hospital in Great Ormond Street, Bloomsbury, which contains 100 beds, and has been erected at a cost of £45,000, of which £35,000 has been already contributed.

We are informed that H.R.H. Princess Mary, Duchess of Teck, has graciously consented to open the new Paddington Green Children's Hospital on Monday, July 1st, at four p.m. Purses containing contributions of £5 and upwards to the rebuilding and extension fund will be received by Her Royal Highness upon the occasion.

Reginald Morton, M.D., F.R.C.S., a Canadian, has been appointed by Lord Brassey, the new Governor of the Colony of Victoria, Australia, surgeon to his well-known yacht, the *Sunbeam*, in which Lord Brassey sails in July next to take up his new office. Dr. Morton, on arrival in Melbourne, enters into partnership with his cousin, Dr. William Morton, who has a large practice in that city.

Through the energy and liberality of Miss Hogg, Brixham has been provided with a new cottage hospital. The building was opened by the Lord Bishop of Caledonia. The hospital will accommodate twelve patients. It is well fitted and of pleasing appearance within and without, and possesses a fine balcony for the benefit of the patients. Miss Hogg will continue Lady Superintendent of the new as of the old hospital.

The new wing of the West Ham Hospital, recently erected out of funds the nucleus of which was supplied by a donation of £2,000 given by Mr. Passmore Edwards, was opened on Saturday afternoon, and the event was made the occasion of an imposing popular demonstration, in which all the friendly societies, political organisations, and temperance associations of the East-end took part. The new wing contains two wards, named respectively the "Eleanor Passmore Edwards" (after Mrs. Passmore Edwards), and "Mr. J. R. Roberts" (after Mr. J. R. Roberts, who has contributed £1,000 to the funds).

In his parting speech at the Middlesex Hospital, Lord Sandhurst emphasised the great interest he took in the affairs of that hospital. Now that he is in India we see how deeply rooted his interest in hospitals is, for amidst the many duties and responsibilities of his new post, we find Lord Sandhurst devoting both time and attention to the institutions where the sick are cared for. At Bombay, where he visited St. George's Hospital, his Excellency's proceedings will serve as an object lesson for official visits, so thorough and searching was his inspection, no detail escaping his observation, the authorities who accompanied him receiving several valuable criticisms. We are not aware what Lord Sandhurst's general impressions of the hospitals of India is, but we hope to find that he agrees with us in thinking the hospital system of the Indian Empire perhaps the most perfect that exists.

